# The Zoonotic Potential of Fungal Pathogens: Another Dimension of the One Health Approach

**DOI:** 10.3390/diagnostics14182050

**Published:** 2024-09-15

**Authors:** Reetu Kundu, Yashik Bansal, Nidhi Singla

**Affiliations:** 1 Department of Cytology and Gynecological Pathology, Post Graduate Institute of Medical Education and Research (PGIMER), Chandigarh 160012, India; reetukundu@gmail.com; 2 Department of Microbiology, MM College of Medical Sciences and Research, Sadopur, Ambala 134007, India; dr.yashikbansal@gmail.com; 3 Department of Microbiology, Government Medical College Hospital, Chandigarh 160030, India

**Keywords:** fungal infection, zoonosis, one health, dermatophyte, sporotrichosis, histoplasmosis, cryptococcosis

## Abstract

Zoonotic diseases are caused by viruses, bacteria, fungi and parasites and they comprise about 75% of all emerging infectious diseases. These can be transmitted via the direct (scratches on skin or animal bites) or indirect mode (through environmental shedding of infectious agent by the infected animal) of transmission. Environmental changes, whether in the form of urbanization, industrialization or destruction of wildlife habitats, lead to more human invasion in wildlife areas, subsequently leading to an increased passage of animals towards human dwellings and more exposure to animals, making humans susceptible to these infections. Climate change is another major factor. Global warming and the evolving thermotolerance of fungi, adapting more to human body temperature than their saprophytic nature, is leading to the emergence of humans as new hosts for fungi. The domestication of animals, rising populations, enhanced tourism, migratory populations, intrusions into wildlife, etc., are other known factors. Zoonotic fungal infections have long been neglected and are now gaining due attention. In this review, we briefly discuss the various aspects currently known for zoonotic fungal infections and bring forth the importance of this particular issue to be addressed in a timely manner.

## 1. Introduction

The World Health Organization (WHO) defines zoonoses or zoonotic infections as those which have natural transmission between vertebrate animals and human beings [1]. Zoonotic diseases are caused by viruses, bacteria, fungi and parasites and they comprise about 75% of all emerging infectious diseases [2]. Zoonotic infections can be transmitted via direct or indirect modes of transmission. Scratches on skin and bites are a direct way of transmitting infection from an infected animal to a human host. When a new host acquires infection through the environmental shedding of the infectious agent by the infected host, this constitutes an indirect mode of spread. For such a transmission to occur-air, water, food or an animal vector act as the vehicle of dispersal [3]. Inter-human transmission is uncommon in zoonotic infections [4].

The WHO has recently declared mpox, a zoonotic disease, to be a Public Health Emergency of International Concern (PHEIC). Although the animal reservoir of the monkeypox virus remains unknown, many rodents like prairie dogs, squirrels, marmots, chinchillas, gerbils, hamsters, etc., can become infected. Zoonotic diseases have the potential to cause pandemics as the infecting agent persists in nature in animal reservoirs [5].

Planet Earth has about 1.5 million species of fungi with only a meagre roughly 300 having causal association with human diseases. Fungi are known to adapt to the environment remarkably. They evolve rapidly under selection pressure due to their capacity for sexual and asexual reproduction, epigenetic change and tolerance to change in their genome, which is small and dynamic with great ecological plasticity, resulting in better thermotolerance and virulence [6,7].

Generally speaking, types of fungal infections in animals can be (1) those without transmission to humans (aspergillosis, mucormycosis, candidiasis, and infections due to melanized fungi); (2) endemic infections with indirect transmission from the environment (coccidioidomycosis, histoplasmosis, paracoccidioidomycosis, and blastomycosis); (3) zoophilic fungal pathogens with near-direct transmission (chytridiomycosis and bat white-nose syndrome); and (4) those which can be directly transmitted from animals to humans (zoophilic dermatophytes, microsporidia, and dimorphic fungi) [8].

The epidemiological triad includes agent, host and environment. Among zoonoses, Claude Bernard’s quote, ‘the terrain is everything, the germ is nothing’, holds true to quite an extent, as the environment is the most significant factor affecting the transmission of the agent from one host (animal) to another, i.e., a human. Environmental changes, whether in the form of urbanization, industrialization or destruction of wildlife habitats, leads to more human invasion into wildlife areas, subsequently leading to increased passage of animals towards human dwellings and more human exposure to animals, making them susceptible to these infections. Climate change is another major factor. Global warming and the evolving thermotolerance of fungi, adapting more to human body temperature than their saprophytic nature, is leading to the emergence of humans as new hosts for fungi. The domestication of animals, rising populations, enhanced tourism, migratory populations, intrusions into wildlife, etc., are other known factors [3].

Another term, sapronoses, is also used for many fungal pathogens, as they are pathogens with zoonotic potential that are able to replicate in and survive on dead organic materials like saprophytes [9]. As per the WHO’s expert committee, sapronoses have been defined as diseases caused by zoonotic pathogens that have a vertebrate host as well as a non-animal reservoir or developmental site like soil, plants, or organic matter. Fungi are basically saprophytic organisms causing infections when host immunity is lowered. The term zoonosis per se does not distinguish between opportunistic infections and true pathogens. Opportunistic fungal infections are caused by fungi which have a preference for environmental niches rather than the host organism. On the contrary, true fungal pathogens affect the host for their survival and multiplication [3].

Overall, the burden of fungal infections is under-estimated, let alone zoonotic infections. The increasing domestication of pets has led to an increasing trend of zoonotic diseases in humans, along with the emergence of newer species causing zoonoses [3]. Hence, continued efforts to increase awareness among the general public, as well as the medical community, are needed. Zoonotic fungal infections have long been neglected and are now gaining due attention. In this narrative review, we briefly discuss the various aspects currently known about zoonotic fungal infections and bring forth the importance of this particular issue to be addressed in a timely manner.

## 2. Methodology

A thorough literature search was carried out using the online databases Cochrane Library, PubMed^®^, Google Scholar, Web of Science™ and Scopus™. The keywords used were zoonoses, fungal zoonoses, neglected fungal zoonoses, fungal infections in animals, emerging mycosis and one health approach. A random search was carried out over the Cochrane Library, PubMed^®^ and Web of Science™ databases, without the application of any filters. Due to the voluminous data without using filters (43,426) across Scopus™ employing the keyword of zoonoses, the search words of fungal and zoonoses were used, yielding 607 results. Subject area, immunology and microbiology were used as filters for further refinement, after which 206 results were obtained. Duplicate results across various databases were excluded. A reference list of the retrieved articles was screened for cross-referencing. Finally, the articles thus obtained were downloaded and evaluated for meeting the scope of this narrative review. A further short-listing of articles was carried out after comprehensive discussion and the pertinent information gathered was utilised for drafting this review.

## 3. Dermatophytoses

Dermatophytes, based on a major natural reservoir, are classified as zoophilic (animals), anthropophilic (humans) or geophilic (soil) [2]. The zoophilic dermatophytes include *Microsporum canis*, *Nannizia nana*, *Nannizia persicolor*, *Trichophyton vanbreuseghemii*, *Trichophyton benhamiae*, *Trichophyton erinacei*, *Trichophyton simii*, *Trichophyton quinckeanum*, *Trichophyton verrucosum*, *Trichophyton equinum* and others [10]. Majorly, four species are responsible for a vast majority of zoonotic dermatophytosis: (1) *Microsporum canis*, which is largely derived from cats and dogs; (2) *Trichophyton verrucosum*, mainly from cattle and Zoophilic species of *T. mentagrophytes*, which are now divided into two species, namely *T. benhamiae* and *T. vanbreuseghemii*; Trichophyton (*Arthroderma*) *vanbreuseghemii*, contracted from cats and dogs; and *Trichophyton (Arthroderma) benhamiae*, derived from guinea pigs [2,10].

They are commonly known as ringworm or tinea [3]. The source of infection in urban areas is stray animals and pets [10]. Zoonotic dermatophytoses or ringworm is a typical example of synanthropic zoonoses having a urban/domestic cycle between animals and humans in contrast to exoanthropic zoonosis, which has a sylvatic cycle [9].

Domestic animals—cats, dogs, goats, pigs, sheep, rabbits, horses, chickens, donkeys, and ducks—serve as reservoirs [2]. New species like *T. erinacei* and *T. benhamiae* have emerged in humans because of the increased domestication of animals [3]. They occur due to direct skin or hair contact with infected animals and fomites. The cutaneous and pilar lesions are generally pruritic. The skin lesions in zoophilic dermatophytosis are known to have pronounced inflammation and a fast progression. Features that indicate towards a zoophilic dermatophytosis are a contact history with pets, vesicular or pustular lesions on exposed areas, and a red rubber ring appearance (one or more rings with a bright, erythematous and oedematous appearance and a central clearing of skin) while post-inflammatory hyperpigmentation and a ring-within-a-ring look, are indicative of anthropophilic cutaneous dermatophytosis [11].

Tinea manuum is an infection of the hand caused globally by the highly prevalent pathogen, *T. rubrum*, followed by *T. mentagrophytes* and *M. canis* [12]. Emerging reports on Tinea manuum caused by *T. erinacei* have surfaced in recent years [13,14,15]. *T. erinacei* has been mostly isolated from hedgehogs. In immunocompromised individuals, a greater virulence of the pathogen and disseminated infection is noted [16]. Recently, *M. canis* was reported as a rare causative agent for finger nail onychomycosis in a teenager with a forthcoming history of contact with her infected pet cat [17]. *M. canis* was identified on cultures from the infected nail and the hair of the pet cat.

The mid twentieth century witnessed a huge human outbreak of dermatophytosis in rural areas of Hungary caused by *T. quinckeanum* [10]. The rodents (mice) had skin lesions as a manifestation of mouse favus. Recently, an epidemic of superficial dermatophytosis caused by *Trichophyton mentagrophytes* has been reported in India [18]. Molecular characterization suggests that *T. indotineae* and *T. interdigitale* act in an anthropophilic manner, while clonal offshoots of *Trichophyton mentagrophytes*, which is likely geophilic, show a shifting of host to domesticated animals [19]. This strain shows frequent resistance to antifungal therapeutic agents, likely due to the overuse of antifungals.

## 4. Sporotrichosis

Sporotrichosis, which is truly polyonymous, is also known as Berumann’s disease, rose gardener’s disease, Schenck’s disease, peat moss disease and subcutaneous or chronic mycotic granulomatous of humans and animals [20]. In many regions of the world it is an emerging zoonotic fungal infection. The causative agent is a thermal dimorphic fungus of the genus *Sporothrix* with a pathogenic clade comprising *S. schenckii, S. globosa*, and *S. brasiliensis* [21]. For a long time, *S. schenckii* was the sole species believed to be the causative disease agent [22]. In the early part of the 21st century, other species like *S. globosa*, *S. mexicana*, *S. luriei* and *S. brasiliensis* were added to the spectrum of causative agents of sporotrichosis based on molecular analysis/multilocus sequence analysis [23,24].

The disease is seen in humans and animals (cats, armadillos, birds, camels, rats, cattle, goats, pigs, chimpanzees, dogs, donkeys, dolphins, hamsters, horses and mules) [20,21]. Almost all cases in human beings with *S. brasiliensis* occur due to traumatic inoculation resulting from a bite, scratch, or contact with the oozing cutaneous lesions of diseased cats [21]. Among all the animals, cats have tremendous zoonotic potential [20]. *S. brasiliensis’* tolerance to the relatively high body temperature of cats may be attributed to the global warming phenomenon [25,26]. *Sporothrix brasiliensis* is also seen among dogs and rats, besides cats [21]. Infected plant material and soil are a point source of infection to which an individual can be exposed [27]. Read and Sperling are given credence for their discovery of the zoonotic potential of *S. schenckii* while probing the disease outbreak attributed to exposure to an infected cat in five individuals [19]. Subsequently, Welsh endorsed the observations initially made by Read and Sperling in their zoonosis update on sporotrichosis [28].

In 1998, the first epidemic of the disease transmitted by cats was reported in Rio de Janeiro, Brazil [21]. The endemic regions include Brazil and Argentina. *S. brasiliensis* is strongly linked with zoonotic transmission involving cats [29]. Over the previous two decades, this has translated into rampant disease outbursts involving felines and humans spanning various regions of Brazil [30]. During a small period of 5 years (2012–2017), 101 human cases of sporotrichosis with 93.4% showing zoonotic transmission were reported in the southern regions of the Rio Grande do Sul state of Brazil [31]. Among these cases, four cases were in the same family who contracted the disease from their domesticated cat. Cat-transmitted sporotrichosis has emerged as a zoonotic epidemic posing major public health concerns in Brazil with the potential to spread to the United States, according to a recent warning from the Center for Disease Control issued early last year [32]. Zoophilic *Sporothrix brasiliensis* has a greater virulence than other species [20]. Notably, this fungus shows resistance to some antifungal agents.

*Sporothrix* infections are usually cutaneous or lymphocutaneous lesions, seen as papules, nodules or ulcers [21]. Pulmonary, osteoarticular and disseminated disease may also be seen [20]. Meningeal involvement, central nervous system manifestations, chronic obstructive pulmonary disease and disseminated disease are more common in persons with Human Immunodeficiency Virus (HIV).

## 5. Histoplasmosis

Histoplasmosis was traditionally known as cave sickness [3]. Once endemic in Ohio state, the St. Lawrence River Valley and the state of Mississippi in United States of America, and in sub-Saharan African regions, the disease has now extended to many other geographical locales [33,34].

The causative agent is the dimorphic fungus *Histoplasma capsulatum*, with varieties being *H. capsulatum var. capsulatum* and *H. capsulatum var. duboisii* [2]. The route of infection is inhalational with air-borne conidia dispersed from the guano of bats and birds enriched with the organism. The causative agent, *Histoplasma*, has been recovered from the gastrointestinal contents and varied organs of both domestic animals and wildlife. Domestic animals include dogs, cats, sheep, cattle and horses, while wildlife chiefly comprises bats and rodents. Other mammalian hosts include bears, monkeys, apes and felines [3]. It has also been isolated from the intestinal contents of a bat, *Nycteris hispida*, dwelling in a cave and having a slit face with long ears and a hairy tail [2]. Tourist activities like exploring caves, bat and bird watching and construction activities or excavation pose imminent danger [3]. The vulnerable population is constituted by farmers, poultry keepers (particularly while cleaning chicken coops, pigeon roosts, barns and lofts), construction workers working in and around old buildings harbouring perching birds and persons engaged in frequent visits to bat-infested caves [2].

The disease manifestations are diverse with pulmonary infection, lymphadenopathy, cutaneous involvement, dissemination to bones and widespread systemic disease [8]. The individual outcome depends on the immunocompetence/immunocompromised status of the host, along with the virulence of the infecting strain and amount of inoculum.

## 6. Malassezia Infection

The year 1925 marked the very first isolation of *Malassezia pachydermatis* from the exfoliated skin of a captive Indian rhinoceros [35]. In the 1950s, it was established as a prominent pathogen causing ear infections among dogs. It is reported that the incidence of otitis externa among dogs is about 10% in primary-care practices and roughly two-thirds of these may be due to *M. pachydermatis*. Fennec foxes, ferrets, pigs and camels can all have otitis associated with *M. pachydermatis*. It may cause seborrheic dermatitis in goats. *Malassezia* yeasts were named as per the Latin nomenclature of warm-blooded vertebrate animals from whom they were isolated, like *M. caprae* (goats), *M. equina* (horses), *M. cuniculi* (rabbits), *M. pstittaci* (parrots) and *M. vespertilionis* (bats). Many breeds of dogs are at risk of developing *Malassezia* dermatitis, namely West Highland White terriers, English setters, shih tzus, basset hounds, dachshunds, American cocker spaniels, poodles, Australian silky terriers and boxers [35].

The zoonotic potential of *Malassezia* was recognized for the first time when a group of low-birth-weight babies in a neonatal intensive care unit receiving lipid emulsions had colonisation by *M. pachydermatis*. This was probably from the hands of health care workers who had contact with pet dogs. These yeasts have the potential to survive on incubator surfaces for a long time [35]. *Malassezia pachydermatis* has also been isolated from a woman with facial granuloma and the skin scrapings and cerumen of her pet dog [36]. Reports of *M. pachydermatis* fungaemia in a 69-year-old with acute myeloid leukaemia on antifungal prophylaxis [37], in an adult patient with multibacillary leprosy hospitalized for *Staphylococcus aureus* bacteremia [38] and in a catheterised patient post radical gastrectomy and chemotherapy for gastric adenocarcinoma [39] have been recorded. Hand contamination from pet dogs with *M. pachydermatis* overgrowth is common. Hence, strict hand hygiene is recommended in persons having contact with them, and more so when they encounter immunocompromised persons to avoid transmitting infection [35].

## 7. Paracoccidioidomycosis

The disease has not been emphatically established as a zoonosis. Its plausible role as a zoonotic fungal disease is considered based on the following facts: (1) its demonstration in armadillos, monkeys and domestic animals; (2) the elusive habitat of the fungus; (3) the rural preference of the disease, which is epidemiologically akin to most zoonotic diseases; and (4) its demonstration of genetically similar strains of the causative agent, *Paracoccidioides brasiliensis*, a thermally dimorphic fungus in infected armadillos and humans in endemic regions [2].

The occurrence of Paracoccidioidomycosis has been reported in domestic and wild animals comprising cows, horses, armadillos, sheep, guinea pigs, monkeys, raccoons, chickens and porcupines [2]. A high frequency of infection has been noted in armadillos, which, epidemiologically, points towards them being a wild host [40]. It has also been reported naturally in cats and dogs, with rural dogs claimed to have a higher infection rate than urban ones [2]. While experimental infection has been achieved in dogs, natural infection is also on record, with lymphadenopathy, hepatosplenomegaly and skinniness [41,42,43,44]. The disease has been associated with rural residency and professional occupations with the possibility of development resulting from exposure to armadillos, bats and coffee plantations [2].

## 8. Talaromycosis

This diseases was previously known as Penicilliosis [3]. It is an emerging infectious fungal disease caused by *Talaromyces/Penicillium marneffei*. It not only causes soft tissue infection but also lytic bone lesions in flat bones. Bamboo rats, *Rhizomys* spp. and *Cannomys* spp. are established enzootic reservoirs [2]. Soil from the burrows of these bamboo rats acts as an important environmental reservoir [45]; however, attempts at isolating the live organism from the soil samples have largely been unsuccessful [46,47]. Two cases with disseminated infection are on record, developing due to exposure to bamboo rats (one involving the killing of bamboo rats and the other due to ingesting bamboo rat meat). Slightly more than a decade ago, dogs were also hinted at as reservoirs [48]. Recently, multilocus genotyping has proven the similarity between the isolates of *T. marneffei* from humans and infecting bamboo rats [47]. Furthermore, the isolates were found to be identical in a few cases. Nonetheless, these observations on *T. marneffei* suggest its zoonotic transmission (direct transmission from rodents to humans), but concrete evidence is yet to be generated.

A summary of common zoonotic fungal infections is given in Table 1.

## 9. Miscellaneous Fungal Diseases with Potential for Zoonotic Transmission to Humans

The zoonotic potential of fungi is not the same and varies among different fungi and from species to species.

### 9.1. Cryptococcosis

This disease, away from the tropical and subtropical regions, is among the deadliest fungal diseases [3]. Two important species in terms of pathogenicity are *Cryptococcus neoformans* (varieties: *C. neoformans* var. *grubii* and *C. neoformans* var. *neoformans*) and *Cryptococcus gattii*. From an ecological perspective, the two species differ, with *C. neoformans* being retrieved from bird droppings and *C. gattii* from eucalyptus trees [1]. *Cryptococcus gattii* has also been recovered from animals like cats, dogs, ferrets, llamas and marine mammals [49]. A broad range of hosts is seen for *C. neoformans*, encompassing lower invertebrates like soil-residing amoebae, cockroaches, nematodes, mites and higher mammals [1].

Cases and outbreaks in domestic and pet animals (cattle, horses, cats and dogs) have been reported [8]. Cats are the most commonly infected animals, where respiratory tract involvement, subcutaneous granulomas and widespread infection may occur. Dogs can also show similar manifestations [1]. The spectrum of cryptococcal infection in humans includes cutaneous, ocular, pulmonary and central nervous system involvement. Meningoencephalitis is commonly observed in immunocompromised individuals, including HIV patients [3].

### 9.2. Emergomycosis

This disease is caused by members of *Emergomyces*. It was earlier known as adiaspiromycosis, for its giant cell variant. *Emmonsia crescens* and *Emmonsia parva* (now *Blastomyces*) are causative pathogens for pulmonary disease in humans and animals [4]. *Emergomyces crescens* is associated with small burrowing mammals, notably Cricetidae and Muridae rodents [3,50]. The world over, they have a wide host range, infecting many species of small mammals [4]. In a study carried out just over two decades ago, in the south-west of the UK, it was shown that 28.7% of free-living wild mammals had a lung infection with *E. crescens* [51]. These observations raise concerns for sporadic infections, which might involve domestic animals and human beings [4]. As of now, irrefutable evidence of emergomycosis being a zoonotic disease is lacking and it is regarded as a potential fungal zoonotic disease, with small rodents and their predators acting as vectors [3].

### 9.3. Trichosporonosis

This disease is caused by the *Trichosporon* species. These are emerging opportunistic fungal pathogens [52]. These fungi dwell in soil, decomposing wooden matter, freshwater and marine environments and wildlife (bats, cattle and fish). Humans are at risk of infection following the intake of contaminated foodstuffs (milk and milk products, honey, seafood and truffles) and due to professional exposure to animals, as is seen with zookeepers and fishers. The isolation of *Trichosporon beigelii* led to speculations that freshwater crayfish were its vector.

### 9.4. Lobomycosis

There are rising reports of lobomycosis, a disease caused by *Lacazia loboi*, in aquatic mammals, particularly dolphins [4]. The disease is documented in two dolphin species: (1) the common bottlenose dolphin (*Tursiops truncatus*) from Europe, Brazil and the Atlantic coast of the United States and (2) the Guiana dolphin (*Sotalia guianensis*) from the Surinam River estuary, South America [53]. In dolphins, verrucous lesions with a tendency for ulceration and plaque formation are described. According to a study utilising photo-identification survey data from over a decade, the prevalence of lobomycosis in bottlenose dolphins from the Indian River Lagoon, FL, USA was 6.8% [54]. Direct contact with infected animals with skin trauma is a possible source of disease transmission. One report of human disease following exposure to a dolphin in an aquarium attendant raises the strong possibility of zoonotic transmission [4].

### 9.5. Entomophthoromycosis

This disease is caused by Entomophthorales (zygomycetes). Causative agents are *Conidiobolus coronatus*, *Conidiobolus incongruous* and *Basidiobolus ranarum* [4]. They are commensals in the gastrointestinal tract of fish, amphibians, insectivorous bats and reptiles. Conidiobolomycosis is primarily seen in horses, dogs and sheep, where ulcerative pyogranulomatous lesions of oral and nasal mucosa are seen. Subcutaneous and invasive infections in humans have been reported. *Basidiobolus ranarum* is generally retrieved from decaying plant matter. Nevertheless, it has been, at times, isolated from the gastrointestinal tracts of amphibians, like frogs and toads; reptiles, like garden lizards and geckos; fish; and mammals, like horses, dogs, insectivorous bats, kangaroos, wallabies and humans [2].

### 9.6. Microsporidia

Microsporidia are now recognised as highly specialized fungi [55,56]. They affect both vertebrate and invertebrate hosts like humans, fish, insects, farm animals and pets [57]. The genera implicated in human and animal disease are *Enterocytozoon*, *Pleistophora*, *Vittaforma*, *Encephalitozoon*, *Trachipleistophora*, *Nosema*, *Brachiola* and *Microsporidium*. Vertical transmission to progeny has been documented in gypsy moth Lymantria, an invertebrate [58]. It has also been reported in vertebrates like rabbits, sheep and primates other than humans. The animal–human food chain allows for disease transmission and emergence in hosts dwelling in aquatic and terrestrial biomes [59]. Furthermore, zoonotic transmission potential through animals which act as reservoirs of infection has been established beyond doubt. Not long ago, experiments established microsporidian *Nosema ceranae* as being highly pathogenic to honeybees (*Apis mellifera*) [60]. Microsporian *Loma salmonae* is plaguing the Canadian salmonid aquaculture industry, causing microsporidial gill disease in salmon [61]. Recently, pertinent transmission and pathogenetic factors responsible for the disease in salmon and trout have been described [62]. Lately, the ultrastructural detailing and molecular characterisation of microsporidians *Thelohania* and *Vairimorpha,* causing microsporidiosis in white-clawed crayfish (a freshwater endangered invertebrate), have been carried out [63].

### 9.7. Blastomycosis

Blastomycosis, now recognized primarily as a disease of canine animals, was first reported by Gilchrist in 1894 in America [64]. It is caused by the thermally dimorphic fungi, *Blastomyces dermatitidis* [65,66]. The fungi gain entry to the body via lungs and cause disseminated infection in immunocompetent as well as immunocompromised individuals, the latter suffering a more severe form of illness. The disease is mainly limited to North America but cases have been described in Africa, the Middle East and India [64].

The prevalence of blastomycosis in dogs is approximately 10 times higher compared to humans and other mammals [67]. Another important observation in a one health context is the reported proximity of dogs affected with the disease to water bodies [68]. Most human infections are reported to be acquired from the environment, but cases do occur as a result of animal (dog) bite or exposure during the autopsy of a deceased animal [64].

### 9.8. Coccidioidomycosis

Coccidioidomycosis is caused by *Coccidioides* and is called San Joaquin Valley fever. Presently, two main species are known—*C. immitis* and *C. posadasii*. It is transmitted through environmental exposure and is acquired via the inhalation of arthrospores. It is endemic in the warm, arid and desert areas of the Western hemisphere. As the attempts to isolate this fungus from soil have not been successful, even in endemic areas, it is speculated that animals act as reservoirs as well as potential disseminators of the fungus in nature [69]. The disease has been reported in animals, namely cattle, cats, horses, dogs, bats and armadillos. Two cases have been reported for zoonotic Coccidioidomycosis, one in a veterinary assistant who developed a localized infection with osteomyelitis as the result of a bite from a cat and the other apparently acquired by inhaling endospores during the necropsy of a horse with disseminated infection [70].

### 9.9. Pneumocystosis

Pneumocystosis is a fungal disease caused by *Pneumocystis* spp. It primarily leads to pulmonary infection in human and animals. As per the literature, each mammalian species of *Pneumocystis* is adapted to its particular host and so may not act as a zoonotic or anthropozoonotic pathogen. Five species have been described, primarily in association with a specific host, and they are as follows: *Pneumocystis jirovecii* (humans), *P. carinii* and *P. wakefieldiae* (brown rats), *P. murina* (mice), and *P. oryctolagi* (rabbits) [71]. Other than this, *Pneumocystis* spp. have been identified in ferrets, sheep, monkeys and in water-borne mammalians like cetaceans [72].

## 10. Others

Aspergillosis has been reported in cats with *A. felis* as a causative agent of sino orbital illness. *Aspergillus* spp. has also been a cause of pouch infections, keratomycosis and pneumonia in horses [8]. Mucormycosis has been reported in wild animals like bison, dolphins and seals. In cattle, mucormycotic ruminitis and lymphadenitis are seen. In dogs, cats and horses, respiratory or gastrointestinal involvement has been reported. Similarly, Candidiasis cases have been reported in cattle, dogs, horses, pigs, etc. Pythiosis is caused by *Pythium insidiosum*. Pythiosis has been seen in horses and dogs, as the animal hosts primarily affected [73,74]. The zoonotic potential of these fungi-like organisms is low. However, considering the increasing compromised immunity in human hosts due to carcinoma, medication, life-style diseases and newer risk factors like chronic liver and kidney disease, the possibility of more transmission of these agents from animals, especially those in the vicinity of humans and raised for by-products like milk, meat, leather, etc., cannot be ruled out. A summary of fungal infections with zoonotic potential is given in Table 2.

## 11. Conclusions

There is no doubt that fungal zoonoses can be a public health problem in the future. The rates are likely to increase further, especially in cases of domestic zoonoses, considering the increasing dependency of humans on animal pets for companionship, emotional and psychological support. It is crucial that this aspect of fungal infections is addressed in a timely manner and handled strategically. Health education and community awareness are most important. Changes in the natural host from animals to humans for zoonotic pathogens can also have serious implications, as this can cause alterations in genetic make-ups, mutations triggering the emergence of drug-resistant species or newer strains among species or clades. Public health measures in the form of due notification, surveillance and uniform policies should be mandated. Critical areas near construction sites, whether for housing, dams, roads, industry, etc., should be identified and preventive measures taken. Until we know the actual burden of this route of transmission and infection, public health measures and prevention strategies at national or international levels will be difficult to plan and implement.

## Figures and Tables

**Table 1:** Summary of zoonotic fungal infections.

Disease	Fungal Pathogen/Species	Animal(s)	Mode of Transmission to Humans—Known/Probable
Dermatophytoses	*Microsporum canis*	All domestic/pet mammals such as cats and dogs, cattle, guinea pigs, hedgehogs and mice	Direct physical contact with an infected animal
	*Trichophyton verrucosum*		
	*Arthroderma vanbreuseghemii*		
	*T. benhamiae*		
	*T. erinacei*		
	*T. quinckeanum*		
Sporotrichosis	*S. brasiliensis*	Cats (most common), dogs, rats, armadillos, horses, camels, cows and pigs	Occupational trauma, scratch or bite from infected animal
Histoplasmosis	*Histoplasma capsulatum*	Bats and birds Also isolated from dogs, cats, sheep, cattle, horses, rodents bears, monkeys, apes and felines	Inhalation of the fungus
Malassezia infection	*M. pachydermatis*	Dogs, cats, cows, pigs, horses, goats, sheep, pigs, rhinoceroses, foxes, ferrets, camels, rabbits, parrots and bats	Direct contact Also, Malassezia spp. can be found as commensals on human skin
	*M. caprae*		
	*M. equina*		
	*M. cuniculi*		
	*M. pstittaci*		
	*M. vespertilionis*		
Paracoccidioidomycosis	*Paracoccidioides brasiliensis*	Armadillos, monkeys and domestic animals (mainly dogs and also cats)	Inhalation of the fungus, compromising the integrity of the skin barrier
Talaromycosis	*Talaromyces marneffei*	Bamboo rats Also reported in dogs and cats	Inhalation of conidia from soil/environment

**Table 2:** Summary of fungal diseases with potential for zoonotic transmission to humans.

Disease	Fungal Pathogen/Species	Animal(s)	Mode of Transmission to Humans—Known/Probable
Cryptococcosis	*Cryptococcus neoformans*	Reported in many birds, reptiles and mammals. Mostly acquired through environments contaminated with bird droppings. Eucalyptus trees act as a source for *C. gatti*	Through inhalation or compromised skin barrier
	*Cryptococcus gatti*		
Emergomycosis	*Emmonsia parva* (now *Blastomyces*)	Squirrels, hedgehogs, raccoons, otters, small mammals such as *Cricetidae* and *Muridae* rodents	Through inhalational route as a result of environments contaminated with material from infected animals
	*Emergomyces crescens*		
Trichosporonosis	*Trichosporon* spp.	Bats, cattle, fish and freshwater crayfish	Occupational exposure among fishermen, consumption or exposure to contaminated animals
	*Trichosporon beigelii*		
Lobomycosis	*Lacazia loboi*	Bottlenose and Guiana dolphins	Inoculation as a consequence of trauma
Entomophthoromycosis	*Conidiobolus coronatus*	Horses, dogs and sheep, frogs and toads, garden lizards and geckos, fish, bats and kangaroos	Through inhalation or traumatic inoculation
	*Conidiobolus incongruous*		
	*Basidiobolus ranarum*		
Blastomycosis	*Blastomyces dermatitidis*	Dogs, horses and cats	Inhalation of conidia
Aspergillosis	*Aspergillus felis*	Cats (*Aspergillus felis*) and horses (*Aspergillus* spp.)	Probably through inhalation of conidia or direct contact
	*Aspergillus* spp.		
Mucormycosis	Various *Mucorales* spp.	Cattle, horses, cats, dogs, bison, dolphins and seals	Probably through inhalation of conidia
Candidiasis	*Candida* spp.	Cattle, dogs, cats, horses, pigs and chickens	Probably through direct contact
Pythiosis	*Pythium insidiosum*	Horses and dogs	Probable exposure to infected animal tissue

## Data Availability

Not applicable.
